# Cyclic loading of tendon fascicles using a novel fatigue loading system increases interleukin-6 expression by tenocytes

**DOI:** 10.1111/j.1600-0838.2011.01410.x

**Published:** 2011-11-03

**Authors:** K Legerlotz, G C Jones, H R C Screen, G P Riley

**Affiliations:** 1 School of Biological Sciences, University of East AngliaNorwich, UK; 2 School of Engineering and Materials Science, Queen Mary University of LondonLondon, UK

**Keywords:** bovine extensor tendon, gene expression, mechanical properties, exercise, collagen, tendinopathy

## Abstract

Repetitive strain or ‘overuse’ is thought to be a major factor contributing to the development of tendinopathy. The aims of our study were to develop a novel cyclic loading system, and use it to investigate the effect of defined loading conditions on the mechanical properties and gene expression of isolated tendon fascicles. Tendon fascicles were dissected from bovine-foot extensors and subjected to cyclic tensile strain (1 Hz) at 30% or 60% of the strain at failure, for 0 h (control), 15 min, 30 min, 1 h, or 5 h. Post loading, a quasi-static test to failure assessed damage. Gene expression at a selected loading regime (1 h at 30% failure strain) was analyzed 6 h post loading by quantitative real-time polymerase chain reaction. Compared with unloaded controls, loading at 30% failure strain took 5 h to lead to a significant decrease in failure stress, whereas loading to 60% led to a significant reduction after 15 min. Loading for 1 h at 30% failure strain did not create significant structural damage, but increased Collagen-1-alpha-chain-1 and interleukin-6 (IL6) expression, suggesting a role of IL6 in tendon adaptation to exercise. Correlating failure properties with fatigue damage provides a method by which changes in gene expression can be associated with different degrees of fatigue damage.

While tendinopathy is a common syndrome in both recreational and elite athletes, its etiology remains poorly understood. Overloading is thought to be a major factor contributing to the development of tendinopathy, causing structural damage and initiating degenerative metabolic changes (Riley, 2004, 2005). Indeed, histological examination of pathological tendon generally reveals significant structural disruption with a notable absence of inflammation, and it was in response to such findings that the generic term ‘tendinopathy’ was adopted in replace of ‘tendinitis’ (Riley, 2004, 2004). However, the actual cellular behavior in the development of tendinopathy remains largely unknown, and inflammation may still play an important role in the early initiation of the disease. Instead of an overstimulation of fibroblasts during fatigue loading, it has also been hypothesized that it might be their understimulation, caused by tissue damage and thus disrupted force transmission, which is initiating a catabolic response. In the long term, this might lead to tissue degeneration and the development of tendinopathy (Arnoczky et al., 2007). Stress deprivation has been shown to increase the expression of the collagen-degrading enzyme matrix metalloproteinase-13 (MMP13, homologue to MMP1 in human) in rat tail tendon, while minimal cyclic loading to 1% strain reduced MMP13 induction, and higher levels of strain (3% and 6%) completely abrogated it (Lavagnino et al., 2003). This suggests that a low level of mechanical loading is essential to maintain matrix integrity and points toward a threshold level below which fibroblasts switch into catabolic mode.

Investigating the early initiation of tendinopathy in an *in vivo* animal model, fatigue loading sufficient to create structural damage (tearing and kinking of collagen fibers) has been shown to be accompanied by an upregulation of the inflammatory mediator interleukin-1β, while moderate loading without evidence of fiber rupture suppressed interleukin-1β expression (Sun et al., 2008). An inflammatory response was also induced by cyclic stretching of cultured tendon fibroblasts. The application of high-level strains in the range of 8% and 12% increased the production of known inflammatory mediators prostaglandin E2, cyclooxygenase-1, and cyclooxygenase-2 (Wang et al., 2003). These studies indicate that acute overloading may induce an inflammatory response in tendon tissue, which in turn may initiate degenerative tendinopathological changes. However, an initial inflammatory response may also be necessary to induce tendon adaptation to exercise, as it is in skeletal muscle; inflammation has been described to be essential for repair and to ensure a positive, adaptive response to trauma in skeletal muscle (Lapointe et al., 2002).

If an inflammatory response is a crucial aspect of the strain response of tendon, it could be hypothesized that the intensity and duration of that response could be critical in controlling the resulting tissue behavior, either maintaining homeostasis or initiating tendinopathy. To understand the initiation and progression of tendinopathy, it is essential to understand the relationship between the structural integrity of tendon tissue and the cellular response. While it is clear that continual fatigue loading of tendon will eventually lead to fibril rupture (Fung et al., 2009), thus compromising the structural integrity of the tendon fascicle, it remains unknown how cellular responses influence and contribute to this process. The aim of our study was to investigate the effect of defined loading or overloading regimes on the mechanical properties of isolated bovine extensor tendon fascicles and to determine early changes in gene expression in response to a specific loading condition. To accomplish this, we have developed a novel multistation cyclic loading system, capable of simultaneously loading up to 16 individual tendon fascicles. The system incorporates 16 individual loading chambers, providing significant versatility for testing multiple loading conditions and enabling individual control of medium supplements, all within a single test run.

## Materials and methods

### Mechanical characterization

**Fig. 1:**
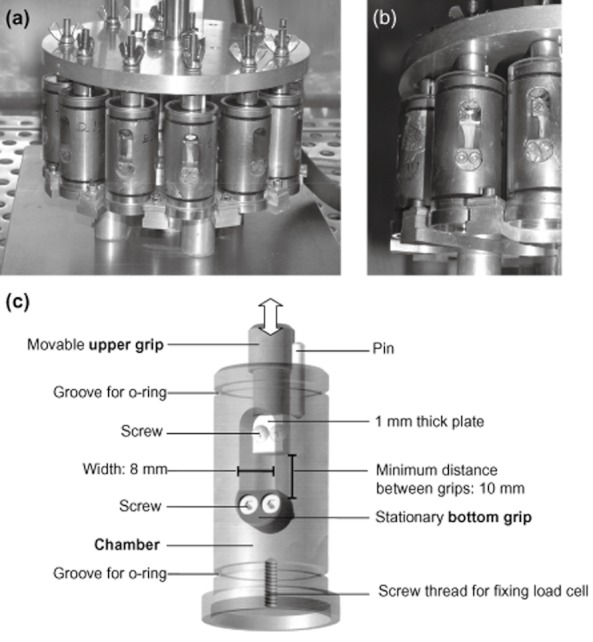
(a) Complete fascicle straining system; (b) close up of an individual loading chamber; (c) drawing of the loading chamber highlighting the components: To secure a fascicle between the upper and the bottom grip, the upper grip is removed from the chamber, the 1-mm thick plate and the screws are removed from the upper grip, and the fascicle is placed on the flat end of the upper grip. The 1-mm thick plate is then dropped back into place and secured with two screws, which are 2 mm apart. The upper grip is then inserted into the chamber, where it is prevented from rotating by a pin at the top of the chamber. The bottom grip is removed and the other end of the fascicle is placed between the two bottom screw holes. The bottom grip is then dropped back into place and secured with two screws. The chamber is filled with medium and sealed with a Plexiglas cylinder. O-rings prevent the chamber from leaking. The chamber is then secured in a rig, which holds up to 16 chambers, and the upper grip is connected to the actuator arm (see image a).

Tendon fascicles were dissected from the bovine foot medial, lateral, or common digital extensor tendon (*n* = 6) by longitudinally cutting with a scalpel along fascicles. The extensor tendons of the bovine foot were selected in preference to the flexor tendons, as the extensor fascicles run in straight lines, and are visible to the naked eye, thus minimizing the likelihood of damage during dissection. All tendons were obtained from young, healthy animals (male steers between 18 and 36 months of age) from a local abattoir and used within 24 h of the animal's death. For each fascicle, the diameter was determined along a 1-cm region in the middle of the fascicle, using a laser micrometer (LSM-501, Mitutoyo, Kawasaki, Japan). The smallest diameter recorded was used to calculate the cross-sectional area (CSA), assuming a circular shape. Each fascicle was secured in an individual custom-made stainless steel loading chamber with a grip to grip distance of 10 mm (Fig. 1). The chambers (up to 16 at a time) were connected to a single actuator arm and secured within a BOSE loading frame (BOSE Corporation, Eden, Prairie, Minnesota, USA), housed in an incubator to maintain samples in 5% CO_2_ at 37°C.

Each chamber was filled with Dulbecco's modified Eagle's medium supplemented with 50 U/mL penicillin and 0.05 mg/mL streptomycin. The fascicles were strained to 2% (0.2 mm) to remove slack (mean point at which force uptake was recorded, as determined from previous experiments), and then loaded at 1 Hz in displacement control, to tensile strains of either 30% or 60% of the strain at failure, for 0 h (control), 15 min, 30 min, 1 h, or 5 h. The strain at failure for 10-mm long samples has been determined in a previous experiment and found to be 22.6% when derived from actuator displacement (Legerlotz et al., 2010). Thus 30% and 60% of strain at failure correspond to 7% and 14% strain, respectively.

Immediately after the fatigue loading, a quasi-static mechanical test was carried out to provide a measure of sample damage. Samples were removed from the BOSE system, still in their test chambers, and each chamber was individually secured in a materials testing machine (Bionix100, MTS, 50N Load cell, MTS Systems Corporation, Eden Prairie, Minnesota, USA). Fascicles were then loaded to failure at a rate of 1 mm/s at room temperature. Force and deformation were both continuously recorded from the Bionix100 at 50 Hz and engineering stress and strain were calculated using the initial CSA and length of the sample. From these data, the loading profile providing the longest fatigue period without significant structural damage was selected (1 h loading at 30% of strain at failure) to investigate gene expression changes with loading.

### Gene expression analysis

Tendon fascicles were dissected from bovine foot digital extensor tendons (*n* = 7). From each animal tendon, one fascicle was frozen directly after dissection as a control, and a second was secured in a loading chamber and loaded for 1 h at 1 Hz to 30% of strain at failure, followed by 6 h of low-level loading at 1 Hz to 10% of the strain at failure. Ten percent of the strain at failure corresponds to 2% strain.

Total RNA was isolated from frozen tissue samples by a modified Tri-Spin protocol as described previously (Ireland et al., 2001). The concentration of RNA was estimated using a NanoDrop spectrophotometer (Wilmington, Delaware, USA). The absorbance ratio A260/A280 was 1.52 ± 0.03 (mean ± SE). Samples were stored at −80°C until further use. Complementary DNA (cDNA) was prepared using SuperScript II (Invitrogen, Paisley, UK) and primed using random hexamers in a total volume of 20 μL according to the manufacturer's instructions. RNA (150 ng) was used for cDNA preparation. The cDNA was stored at −20°C. mRNA levels of 11 genes were quantified using quantitative real-time polymerase chain reaction (qRT-PCR): collagen type I alpha chain 1 (COL1A1), collagen type I alpha chain 2 (COL1A2), connective tissue growth factor (CTGF), insulin-like growth factor 1 (IGF1), interleukin 6 (IL6), interleukin 6 receptor (IL6R), transforming growth factor beta 1, transforming growth factor beta 2, transforming growth factor beta 3 (TGFb3), 18S ribosomal RNA (18S), and glyceraldehyde-3-phosphate dehydrogenase (GAPDH). 18S and GAPDH were used as housekeeping genes. Specific primers were designed using Primer Express 1.0 software (Applied Biosystems, Warrington, UK) and used with either specifically designed fluorogenic probes, a universal probe, or SYBR green (Table 1). To control against amplification of genomic DNA, primers were designed, where possible, so that amplicons crossed exon boundaries. Primers were also checked for gene specificity using primer BLAST. When using SYBR green with the primers, melting curves were analyzed to ensure that only one product was amplified. The relative quantification of genes was performed using the 7500 Real Time PCR System (Applied Biosystems). Each reaction was performed in 25 μL and contained the equivalent of 0.75 ng of reverse-transcribed RNA, 50% TaqMan 2 × PCR Master Mix (Applied Biosystems), 100 n*M* each of the forward and reverse primer, and 200 n*M* of probe, respectively 0.3 μL SYBR green per well when no probe was used. Conditions for the PCR were 2 min at 50°C, 10 min at 95°C, and then 40 cycles, each consisting of 15 s at 95°C and 1 min at 60°C. The cycle number [termed the cycle threshold (Ct)] at which amplification entered the exponential phase was determined. To present the relative gene expression, the comparative Ct method was used according to the fomula: 2^−ΔΔCt^ = [(Ct gene of interest_fatigue loaded − Ct housekeeping_fatigue loaded) − (Ct gene of interest_control − Ct housekeeping_control)] (Schmittgen & Livak, 2008).

**Table 1:** Primers and probes used for quantitative real-time PCR

Gene name	Primers and probes
18S	Forward: 5′-TGC GGC TTA ATT TGA CTC AAC A-3′
	Reverse: 5′-CGA GAA AGA GCT ATC AAT CTG TCA AT-3′
	Used with SYBR green
GAPDH	Forward: 5′-ATG GAA AGG CCA TCA CCA TCT-3′
	Reverse: 5′-CCA CTA CAT ACT CAG CAC CAG CAT-3′
	Probe: 5′-FAM-CGA GAT CCT GCC AAC ATC AAG TGG G-TAMRA-3′
COL1A1	Forward: 5′-GCC TGG TCA GAG AGG AGA AAG A-3′
	Reverse: 5′-CCT TGT TTG CCG GGT TCA C-3′
	Probe: 5′-FAM-TTC CCT GGT CTT CCT G-TAMRA-3′
COL1A2	Forward: 5′-GGT AGG AGA AAC TAT CAA CGG TGG TA-3′
	Reverse: 5′-AAG GCA AGT TGG GTA GCC ATT-3′
	Used with SYBR green
CTGF	Forward: 5′-GGA GGA GAA CAT TAA GAA AGG CAA A-3′
	Reverse: 5′-CAG CCA GAA AGC TGA AAC TTG ATA-3′
	Used with SYBR green
IGF1	Forward: 5′-TGT GAT TTC TTG AAG CAG GTG AA-3′
	Reverse: 5′-AGC ACA GGG CCA GAT AGA AGA G-3′
	Used with SYBR green
IL6	Forward: 5′-CCA GAG AAA ACC GAA GCT CTC A-3′
	Reverse: 5′-CTC ATC ATT CTT CTC ACA TAT CTC CTT T-3′
	Used with SYBR green
IL6R	Forward: 5′-TCC CCA GAA GGA GAA CTG G-3′
	Reverse: 5′-AGG CAA TGC TGA TTT CAC AA-3′
	Used with universal probe #29 (Roche, Burgess Hill, UK, Cat. No. 04687612001)
TGFb1	Forward: 5′-CAC GTG GAG CTG TAC CAG AAA TAT-3′
	Reverse: 5′-CAA CTC CAG TGA CGT CAA AGG A-3′
	Used with SYBR green
TGFb2	Forward: 5′-GCC GAG TTC AGA GTC TTT CGT T-3′
	Reverse: 5′-GAT TTG AGA ATC TGA TAC AGC TCG AT-3′
	Used with SYBR green
TGFb3	Forward: 5′-TTA CTG CTT CCG CAA TTT GGA-3′
	Reverse: 5′-CCT TAG GTT CAT GGA CCC ATT TC-3′
	Used with SYBR green

18S, 18S ribosomal RNA; COL1A1, collagen type I alpha chain 1; COL1A2, collagen type I alpha chain 2; CTGF, connective tissue growth factor; GAPDH, glyceraldehyde-3-phosphate dehydrogenase; IGF1, insulin-like growth factor 1, IL6, interleukin 6; IL6R, interleukin 6 receptor; TGFb1, transforming growth factor beta 1; TGFb2, transforming growth factor beta 2; TGFb3, transforming growth factor beta 3.

### Statistical analyses

The decrease in failure stress with fatigue compared with an unloaded control was analyzed using a one-way analysis of variance. Differences in failure stress between fatigue loading at 30% or 60% of strain at failure were detected using an independent *t*-test. To detect differences in gene expression between fatigue loaded and control specimens, a Wilcoxon test was performed on the 2^−ΔCt^ values. For all statistical tests, significance was established at *P* ≤ 0.05.

## Results

**Fig. 2:**
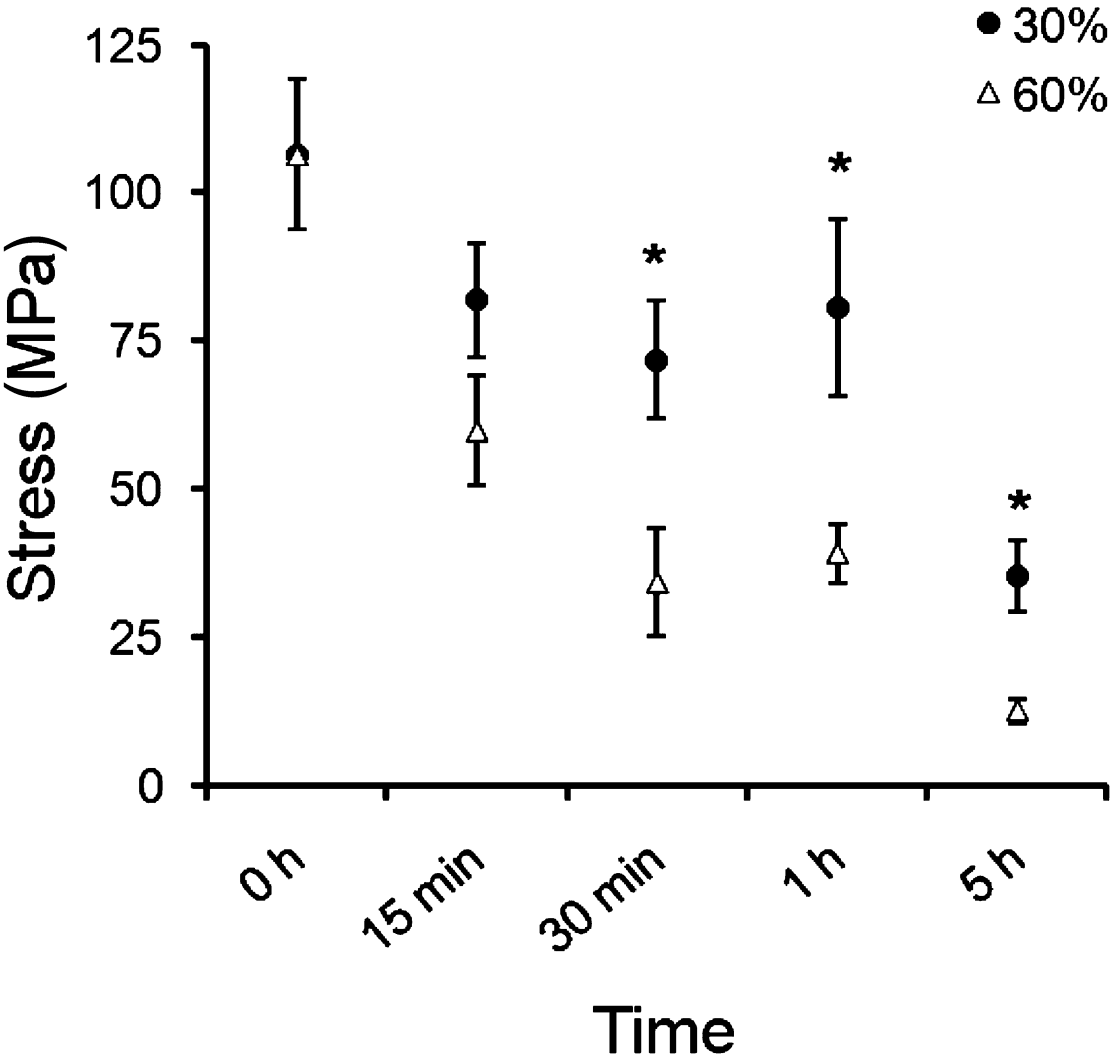
Quasi-static failure stress of samples after previous fatigue loading. Control samples are presented as 0 h loading. Filled circles show samples subjected to 30% of the strain at failure. Triangles show samples loaded to 60% of the failure strain. *Significant difference between 30% and 60% loading groups (independent *t*-test). Data are presented as mean ± SE. (*n* = 6).

Fatigue loading of tendon fascicles to 60% of the strain at failure induced greater reductions in quasi-static failure stress compared with loading to 30% strain at failure at all time points, with significant differences between the two groups after 30 min, 1 h, and 5 h loading (Fig. 2).

Compared with unloaded controls, loading to 30% of strain at failure took 5 h to lead to a significant decrease in failure stress of 67%, whereas loading for 15 min, 30 min, or 1 h resulted in small and nonsignificant reductions in failure stress. Loading to 60% of the strain at failure led to a significant 44% reduction in the failure stress after only 15 min. After 5 h, failure stress dropped to 12% of that reported in the unfatigued specimens.

**Fig. 3:**
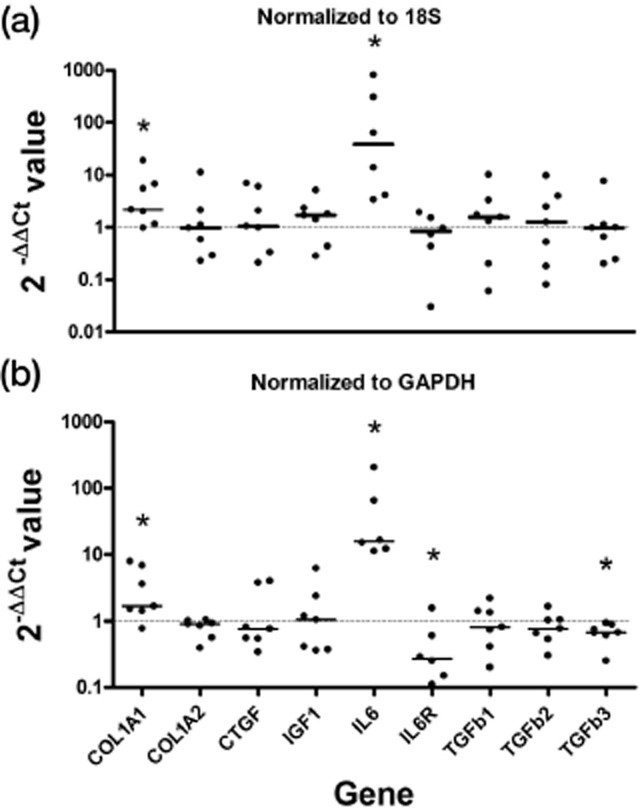
Fold change in mRNA expression of the fatigue-loaded specimens relative to control specimens as represented by the 2^−ΔΔCt^ values normalized to 18S (a) or GAPDH (b). The median is represented by the line through the group of individual data points. The dashed line indicates a value of 1, representing the no-change level. *Significant difference between fatigue-loaded and control specimens (Wilcoxon test) (*n* = 7). 18S, 18S ribosomal RNA; COL1A1, collagen alpha chain 1; COL1A2, collagen alpha chain 2; CTGF, connective tissue growth factor; GAPDH, glyceraldehyde-3-phosphate dehydrogenase; IGF1, insulin-like growth factor 1, IL6, interleukin 6; IL6R, interleukin 6 receptor; TGFb1, transforming growth factor beta 1; TGFb2, transforming growth factor beta 2; TGFb3, transforming growth factor beta 3.

Investigating gene expression of samples subjected to 30% of strain at failure for 1 h highlighted a significantly higher expression of COL1A1 and IL6 in fatigue-loaded specimens compared with controls, when normalized to either housekeeping gene. The median change compared with the control was twofold for COL1A1 with either housekeeping gene, and 16-fold and 38-fold for IL6 when normalized to GAPDH and 18S, respectively. IL6R and TGFb3 expression appeared to reduce significantly with loading, but only when normalized to GAPDH (Fig. 3).

## Discussion

The novel fatigue loading system successfully generated mechanical damage within the tendon fascicles, characterized by the reduction in quasi-static stress to failure in these samples. Quasi-static mechanical characterization of samples post fatigue indicated that loading at 30% of the failure strain took 5 h to generate structural damage, hence a regime of 1 h loading at 30% of the strain at failure, which did not create significant changes to the gross mechanical properties, may approximate to a more physiological exercise environment. This minimal damage protocol was selected to investigate gene expression changes with loading. The major changes were an increase in COL1A1 and IL6 mRNA expression, suggesting that this loading regime is sufficient to create an exercise adaptation response.

Type I collagen is the main constituent of tendon and responsible for its high tensile strength. An increase in COL1A1 expression in response to exercise or loading is most likely necessary to maintain the strength and structural integrity of the tissue. In the current study, we have shown a twofold increase in COL1A1 expression in response to loading. Although similar changes in collagen expression have been reported previously, there is substantial variability between studies, as collagen expression and synthesis in response to loading seem to be affected by a variety of factors including loading magnitude, loading duration, time point of sampling, and gender (Langberg et al., 1999; Miller et al., 2005, 2007; Heinemeier et al., 2007; Legerlotz et al., 2007; Maeda et al., 2007; Maeda et al., 2009; Sullivan et al., 2009; Andersen et al., 2011).

Previous studies at the protein level have shown an upregulation in collagen synthesis, assessed by [^3^H]-proline incorporation, following 5% cyclic tensile strain of isolated rat tail tendon fascicles after 24 h (Screen et al., 2005; Maeda et al., 2007), while collagen synthesis was inhibited at earlier time points (10 min and 6 h) (Maeda et al., 2007). In the latter study, the levels of applied strain were broadly similar to the level of strain used in the present study. Taking the grip-to-grip distance of 20 mm in Maeda's study into account, the applied strain of 5% (= 1 mm deformation) corresponds to 43% of the strain at failure, or to 34% if 0.2 mm is accounted for slack.

It might be assumed that changes in collagen synthesis would be preceded by changes in collagen gene expression. However, COL1A1 mRNA expression remained unchanged (Maeda et al., 2009) while collagen synthesis, as determined by [^3^H]-proline incorporation, increased (Maeda et al., 2007), following cyclic tensile strain of a 3% amplitude superimposed on a 2% static strain applied to rat tail tendon fascicles over a 24-h period. Exercise has even been shown to lead to a decrease in collagen mRNA expression; when 12 recreationally active men and women performed a regime of resistance exercises consisting of unilateral knee extensions (three sets of 10 repetitions at 70% of the one repetition maximum), COL1A1 mRNA expression in the patellar tendon decreased significantly 4 h post-exercise and returned to resting levels after 24 h (Sullivan et al., 2009). In contrast, more repetitions of a similar type of exercise (1 h of one-legged kicking at 67% of the workload maximum) led to an increase in the fractional rate of collagen-1 synthesis after 6 h, when directly measured in biopsies from the patellar tendon after tracer infusion (Miller et al., 2005), indicating that collagen synthesis induction might depend on a combination of both the duration and intensity of the loading stimulus. Effects of loading intensity and duration can also be seen when comparing studies in the rat Achilles tendon, in which 4 days of concentric, eccentric, or isometric training through electric muscle stimulation increased COL1A1 mRNA measured 24 h after the last training bout (Heinemeier et al., 2007), but 12 weeks of voluntary running or strength training did not induce any changes (Legerlotz et al., 2007).

While collagen synthesis and mRNA expression are highly variable depending on the specific experimental conditions, the response of IL6 to exercise is remarkably unambiguous. Exercise is known to increase plasma IL6 levels. While the exercising muscle is traditionally thought to be the major site of IL6 production (Steensberg et al., 2000), a dramatic increase in the interstitial concentration of IL6 has been found in the peritendinous tissue around the human Achilles tendon after running, pointing toward tenocytes as a possible source of IL6 synthesis (Langberg et al., 2002). However, it is difficult to isolate metabolic changes *in situ*, and the adipose tissue situated in the region, like the Kager's fat pad anterior to the Achilles tendon, might have contributed to the measured increase in IL6. Adipose tissue has been shown to respond to exercise, with mRNA levels of IL6 increasing 33-fold 90 min after a 3 h bicycle exercise in the abdominal adipose tissue in healthy young men (Keller et al., 2003). Indeed, in this study the induction of IL6 expression was more pronounced in the fat tissue compared with muscle tissue taken from the vastus lateralis, which only increased ninefold at its peak immediately after exercise.

By isolating the tendon response, our study confirms that tendon tissue does express IL6 in response to exercise with a marked increase compared with very low pre-exercise levels. However, as IL6 has been shown to have both pro- and anti-inflammatory functions (Fisman & Tenenbaum, 2010), it could play a role in either tendon adaptation or the development of tendinopathy. Increased IL6 levels might facilitate tendon adaptation by regulating healing processes, as tendon healing has been shown to be impaired in IL6 knockout mice (Lin et al., 2006). IL6 might also affect the tendon structure, as the cross-sectional area of the patellar tendon was decreased in IL6 knockout mice (Lin et al., 2005). However, the maximum stress was not affected and the modulus even increased while the fiber distribution remained unchanged (Lin et al., 2005). Interestingly, the changes in tendon mechanical properties or tendon healing seen with IL6 could be a result of changes in collagen metabolism, as IL6 has been shown to inducecollagen synthesis. When recombinant IL6 was infused in young men, the concentration of a procollagen marker rose in the peritendinous space around the Achilles tendon, no matter if they performed a 1 h running exercise prior to infusion or rested, while exercise alone did not induce collagen synthesis (Andersen et al., 2011). However, while a number of studies are consistent with IL6 playing a positive role in tendon adaptation, IL6 has also been shown to induce fibroblast proliferation (Mihara et al., 1995). As hypercellularity has often been described as a symptom of tendinopathy (Riley, 2004, 2005, 2004; Lian et al., 2007), this may be indicative of a negative IL6 effect in tendon.

Correlating quasi-static failure properties with fatigue damage provides a novel method by which the extent of fatigue damage can be quantified, and thus enable changes in gene expression to be associated with different degrees of sample fatigue. Although efforts were made to keep the fascicles in an environment which mimics the physiological conditions as closely as possible (e.g. kept at 37°C at controlled O_2_ and CO_2_ in serum-free medium), it is known that simply working in an *in vitro* environment can influence gene expression and mechanical properties (Screen et al., 2006; Leigh et al., 2008). However, *in vitro* loading provides a range of alternative benefits over *in vivo* testing, enabling both the applied load and the resulting tissue damage to be controlled and characterized precisely. Indeed, unloading of tendon tissue has been shown to induce changes in gene expression, particularly MMP 3 and MMP 13 (Leigh et al., 2008). Accordingly, the effects of unloading were avoided in our study by subjecting fascicles to low-level loading (at 10% of the strain at failure) after the 1 h loading period of interest, providing time for the cells to respond to the loading stimulus. *In vitro* fatigue loading of tendons (Fung et al., 2009), tendon cells (Lavagnino et al., 2003), or fascicles (Maeda et al., 2007) is frequently carried out under displacement control (keeping the strain level constant) as this generally enables tighter control over loading conditions. The significant levels of inter- and intra sample variability suggest that it may be advisable to apply fatigue loading under displacement rather than load control, as we have shown previously that tendon fascicle strain is a less variable parameter than stress (Legerlotz et al., 2010). Still, displacement control is likely to be in contrast with *in vivo* conditions as the stress experienced by the tissue will drop during loading in displacement control due to tissue relaxation. This force drop is unlikely to occur in the *in vivo* situation given constant body mass and speed. To provide more versatility and allow future experiments to be run in load control, the fatigue loading system is currently being adapted and each chamber is fitted with an individual load cell.

## Perspectives

The involvement of IL6 in tendon healing, its induction with exercise and its role in stimulating collagen synthesis, together suggest that IL6 plays an important role in tendon metabolism in response to exercise. In light of recent findings describing the negative effect of anti-inflammatory medication on collagen synthesis after exercise (Christensen et al., 2011), a modest inflammatory response, in which IL6 might be involved, may be necessary for tendon adaptation. Recombinant IL6 blockers have been successfully applied to treat rheumatoid arthritis (Choy et al., 2002), and this study indicates the need for further investigation into IL6, as a potential target for therapeutic intervention in tendinopathy.
